# Tuberculosis from the perspective of men and women^*^

**DOI:** 10.1590/1980-220X-REEUSP-2022-0137en

**Published:** 2022-11-07

**Authors:** Talina Carla da Silva, Mayrla Lima Pinto, Giovanna Mariah Orlandi, Tânia Maria Ribeiro Monteiro de Figueiredo, Francisco Oscar de Siqueira França, Maria Rita Bertolozzi

**Affiliations:** 1 Universidade de São Paulo, Escola de Enfermagem, Departamento de Enfermagem em Saúde Coletiva, São Paulo, SP, Brazil.; 2 Universidade Federal da Paraíba, Hospital Universitário Lauro Wanderley, João Pessoa, PB, Brazil.; 3 Universidade Estadual da Paraíba, Faculdade de Enfermagem, Departamento de Enfermagem, Campina Grande, PB, Brazil.; 4 Universidade de São Paulo, Faculdade de Medicina, Departamento de Moléstias Infecciosas e Parasitárias, São Paulo, SP, Brazil.

**Keywords:** Tuberculosis, Treatment Adherence and Compliance, Gender Identity, Tuberculosis, Cumplimiento y Adherencia al Tratamiento, Identidad de Género, Tuberculose, Cooperação e Adesão ao Tratamento, Identidade de gênero

## Abstract

**Objective:**

To analyze aspects related to the experience of tuberculosis from the perspective of men and women with tuberculosis.

**Method:**

Qualiquantitative cross-sectional study. Patients with tuberculosis in the city of Campina Grande-PB were interviewed through a semi-structured questionnaire between September/2017 and January/2018. Discourse Analysis and Chi-Square test were performed.

**Results:**

Sixty-three subjects were interviewed, of which 34 (54.0%) were men. There was an association of the category gender with level of education (p = 0.004), work activity (p = 0.023), time spent on activities outside the home (p = 0.013), and time spent on activities at home (p = 0.001). The analysis of the statements specially revealed that men perceive their role as the family’s main provider and the women with a social role of caregiver, often postponing the search for a health care due to fear of not being able to perform this role in the family and/or in society.

**Conclusion:**

The weakness caused by the disease and the long duration of treatment changed the interviewees’ routine, causing suffering and frustration, with consequences in the performance of social roles within the family and in society, constituting a barrier to adherence to tuberculosis treatment.

## RESUMO

**Objetivo:**

Analisar aspectos relacionados à vivência da tuberculose na perspectiva de homens e mulheres portadores de tuberculose.

**Método:**

Estudo transversal qualiquantitativo. Entrevistou-se, por meio de questionário semiestruturado, pacientes com tuberculose no município de Campina Grande-PB, entre setembro/2017 e janeiro/2018. Realizou-se Análise de Discurso e teste Qui-Quadrado.

**Resultados:**

Entrevistaram-se 63 sujeitos, sendo 34 (54,0%) do sexo masculino. Evidenciou-se associação da categoria gênero com escolaridade (p = 0,004), atividade de trabalho (p = 0,023), tempo despendido em atividades fora do domicílio (p = 0,013) e tempo despendido em atividades no domicílio (p = 0,001). A análise dos depoimentos revelou, principalmente, que o homem percebe seu papel como provedor principal da família e, a mulher, no papel social de cuidadora, adiando muitas vezes a busca por um serviço de saúde com receio de não conseguir exercer esse papel na família e/ou na sociedade.

**Conclusão:**

A debilidade causada pela doença e a longa duração do tratamento mudaram a rotina dos entrevistados, causando sofrimento e frustração, com decorrências no desempenho dos papéis sociais no interior da família e na sociedade, constituindo-se em barreira na adesão ao tratamento da tuberculose.

## RESUMEN

**Objectivo:**

Analizar aspectos relacionados con la experiencia de la tuberculosis desde la perspectiva de hombres y mujeres con tuberculosis.

**Método:**

Estudio transversal cualicuantitativo. Fueron entrevistados pacientes con tuberculosis del municipio de Campina Grande-PB, por medio de cuestionario semiestructurado, entre septiembre/2017 y enero/2018. Se realizaron Análisis del Discurso y Chi-Cuadrado.

**Resultados:**

Sesenta y três sujetos fueron entrevistados, de los cuales 34 (el 54,0%) eran del sexo masculino. Hubo asociación de la categoria género con escolaridad (p = 0,004), actividad laboral (p = 0,023), tiempo dedicado a actividades fuera del domicilio (p = 0.013) y tiempo dedicado a las actividades em el domicilio (p = 0,001). El análisis de las declaraciones reveló, principalmente, que el hombre percibe su rol como principal proveedor de la familia y, la mujer, em el rol social de cuidadora, postergando muchas veces la búsqueda de un servicio de salud por temor a no poder realizar ese rol en la familia y/o en la sociedad.

**Conclusión:**

La debilidad provocada por la enfermedad y la larga duración del tratamiento modificaron la rutina de los entrevistados, provocando sufrimiento y frustración, con consecuencias en el desempeño de los roles sociales en la familia y en la sociedad, constituyendo una barrera para la adherencia al tratamiento de la tuberculosis.

## INTRODUCTION

Tuberculosis (TB) remains being an important public health problem, affecting mainly people with immunological impairment and social conditions that show vulnerabilities. Although the disease no longer appears in the world ranking among the 10 leading causes of death, in which chronic non-communicable diseases predominate, it remains an important infectious disease in low- and middle-income countries. In these countries, TB occupied, respectively, the eighth and seventh positions among the causes of death in 2019, evidencing the social determination of the disease and its relationship with the increase in social inequalities^(1,2)^.

In fact, around 1.2 million people died in 2019 from the disease^(3,4)^. Although, proportionally, it has affected more men than women, among infectious diseases, it is the disease that has led women to death the most. Assuming that TB is closely related to social inequities and that women constitute the majority of the population in poverty, worldwide, the vulnerability of this population group is highlighted^(3,5)^.

Several aspects make it difficult to control TB, such as: increasing resistance to drugs, use of alcohol and drugs, smoking, association with HIV, failures, and delay in diagnosis and mistakes in the treatment follow-up by the patient. Particularly in men, there is a prevalence of smoking and use of alcohol and drugs, which may contribute to the high burden of disease in this group^(3)^. Early detection of TB is one of the global control strategies established by the World Health Organization (WHO), and its scope also depends, among others, on the quality of health services, the involvement of professionals, and the patient’s perception about the disease, access to health services^(3,6)^. The WHO points out that women access diagnostic and treatment services more frequently than men, who, paradoxically, have a higher burden of disease^(3)^, as a result of contexts of vulnerability that may be different from those related to women.

However, it is not a matter of minimizing such differences, reducing them to sex, which is related to the physiological aspects of the human being – to the biologically determined characteristics that distinguish men from women. It is about broadening the interpretation, including the gender perspective that, despite the complexity in its definition, can be understood as a product of social, cultural, and historical relations, which establish the roles played by men and women^(7)^. The WHO considers gender a potent determinant of health that interacts with the family structure, social support, behaviors, influencing the perception of men and women of the health-disease process, making them even more vulnerable to illnesses, by also articulating it to other health determinants, such as social status, position in the economy, access to resources, among others^(8,9)^.

In the literature, the influence of the category gender on adherence to TB treatment has not been analyzed. Studies on TB and gender are generally limited to addressing differences between the sexes^(9,10)^. However, admitting gender as a relevant topic for public health as it constitutes a barrier to the control of the disease, research on gender and adherence to TB treatment are relevant to improve the understanding of the dynamics of this disease in the community^(11)^. From this perspective, the study aimed to analyze aspects related to the experience of tuberculosis from the perspective of men and women with TB.

## METHOD

### Design of Study

A descriptive-exploratory cross-sectional study was carried out, with a qualiquantitative approach^(12)^.

### Population

The sample consisted of patients who underwent treatment for TB in 2016.

### Local

The study was developed in the city of Campina Grande-PB. When it comes to care for people with TB, the Municipality follows the recommendations of the National Tuberculosis Program (*PNCT*), having decentralized treatment monitoring to the Family Health Strategy teams.

### Selection Criteria

Data were initially extracted from the Brazilian government Notifiable Diseases Information System (*SINAN*). Inclusion criteria were: being 18 years of age or older, residing in the study location, presenting the complete address and preserved ability to understand and communicate verbally. The exclusion criteria were: people who, during the period of data collection, were incarcerated in the prison system; those who died; when there was a transfer, diagnostic change, address change; people residing in rural areas; and in the case of duplicate data in the *SINAN* system. Thus, the sample consisted of 63 cases.

### Data Collection

Data collection from the patients took place between September 2017 and January 2018, through an interview held at the residence of the study participants. The addresses were made available by the Health Department of the Municipality where the study was carried out and confirmed with the Basic Health Units where the patients underwent treatment. A semi-structured questionnaire containing two parts guided the interviews. The first part sought to identify the participants’ sociodemographic information: age, education, marital status, people in the household, work status, hours spent on household activities and activities outside the household, in addition to the treatment status. The second part contained the guiding questions that allowed the participants’ free speech. Prior to data collection, a pilot test was carried out with 10 TB patients to improve the instrument, the results of which were not included in the study. The interviews were audio-recorded and transcribed in full and this material constituted the raw material for the statements analysis.

### Data Analysis and Treatment

Regarding the landmark that led to the analysis and interpretation of the data, the concept of *adherence* was adopted^(13)^. In it, adherence is not reduced to personal volition, but is related to the place occupied by the individual in society, to their perception of the health-disease process and to the organization of health work processes^(13)^. Regarding the concept of *gender*, it consists of the social relationships between men and women and the construction of the roles of men and women in society, which end up influencing their behavior in all aspects of life^(8)^.

Quantitative data were analyzed according to frequency and chi-square test (*x* ^*2*^) or Fisher’s exact, when necessary, establishing a confidence interval of 95% and a significance of 5%. Stratification by sex was performed to assess differences between men and women. Quantitative data were systematized in Excel and processed in SPSS (*Statistical Package for Social Science*) version 13.0.

The statements were submitted to the adapted Discourse Analysis technique^(14)^, which allowed the emergence of themes (the abstract elements portraying the reality perceived by the senses) and figures (the concrete elements present in the speeches, words that represent reality)^(14)^. Several readings of the statements were carried out, in an attempt to reach the deeper structure of the texts and to build the categories. With this, the nuclei of meaning related to the object of study were revealed. Participants’ confidentiality was maintained and each participant was duly identified: men – H and women – M.

### Ethical Aspects

The present study complies with Resolution 466/2012 and was approved by the Research Ethics Committee, under number 2.240.525. All participants signed the Free and Informed Consent Form.

## RESULTS

Of the 63 subjects interviewed, 34 (54.0%) were male. In Table 1 it can be observed that age is very similar among both groups; men had low or no level of education (p = 0.004), worked (p = 0.023), and had more time dedicated to extra-domestic activities (p = 0.013). The female participants had higher levels of education (p = 0.004), worked (p = 0.023), and devoted more time to activities at home (p=0.001).

**Table 1.** Distribution of study participants according to personal and social characteristics – Campina Grande, PB, Brazil, 2016.

Variables	Total		Women		Men		χ^2^ test p-value
	No.	%	No.	%	No.	%	
**Age group (years)**							p = 0.984
19 |---- 30	14	22.2	7	24.1	7	20.6	
30 |---- 41	12	19.0	5	17.2	7	20.6	
41 |---- 52	16	25.4	8	27.6	8	23.5	
52 |---- 63	12	19.0	5	17.2	7	20.6	
63 |----| 74	9	14.3	4	13.8	5	14.7	
**Education – last grade studied**							p = 0.004*
Not literate	26	42.6	6	21.4	20	60.6	
Finished elementary school	4	6.6	0	0.0	4	12.1	
Finished elementary school	11	18.0	7	25.0	4	12.1	
Unfinished high school	9	14.8	6	21.4	3	9.1	
Finished high school	4	6.6	4	14.3	0	0.0	
Unfinished higher degree	5	8.2	3	10.7	2	6.1	
Finished higher degree	2	3.3	2	7.1	0	0.0	
**Marital status**							p = 0.320
Common law marriage	9	14.5	3	10.7	6	17.6	
Married	21	33.9	8	28.6	13	38.2	
Single	25	40.3	12	42.9	13	38.2	
Divorced	4	6.5	2	7.1	2	5.9	
Widow(er)	3	4.8	3	10.7	0	0.0	
**Number of people in the household**							p = 0.310
1 to 2	20	31.7	12	41.4	8	23.5	
3 to 4	27	42.9	11	37.9	16	47.1	
5 to 7	16	25.4	6	20.7	10	29.4	
**Work situation**							p = 0.023*
Working	33	53.2	9	33.3	24	68.6	
Unemployed	12	19.4	6	22.2	6	17.1	
Leave	4	6.5	2	7.4	2	5.7	
Retired	6	9.7	3	11.1	3	8.6	
Student	5	8.1	5	18.5	0	0.0	
Work at home	2	3.2	2	7.4	0	0.0	
**Hours of home activities**							p = 0.001*
0 |---- 4 h	37	58.7	10	34.5	27	79.4	
4 |---- 8 h	15	23.8	11	37.9	4	11.8	
8 |----| 12	11	17.5	8	27.6	3	8.8	
**Time spent outside the home**							p = 0.013*
0 |---- 4 h	27	42.9	18	62.1	9	26.5	
4 |---- 8 h	13	20.6	5	17.2	8	23.5	
8 |----| 12	23	36.5	6	20.7	17	50.0	
**Treatment status**							p = 0.688
Cure	49	84.5	24	88.9	25	80.6	
Abandonment	3	5.2	1	3.7	2	6.5	
Progress	6	10.3	2	7.4	4	12.9	

(+) Chi-square test. Significant result: (*) p-value < 0.05. (a) Fisher’s exact test (expected frequency < than 5). Significant result: (*) p-value < 0.05. (-). (b) related variables. For this analysis, missing values were excluded.

Regarding cure, it was higher among women (88.9%) and abandonment was higher in the group of men (6.5%) (Table 1).

With regard to the analysis of the statements, it was found that they reflect the role historically constructed and socially reproduced for both sexes. Men understand their participation in the family mainly as “providers”: *I believe that I am the strongest point (…)* (HFF9), responsible for ensuring the family’s livelihood (HFF9; HH8; HI9; HO9; HU4; HDD3; HDD19).

On the other hand, the woman reveals herself as a “caregiver”: *My role is always to take care of them (…)* (FK7); housewife and central support of the family (FA5; FB8; FE4; FG11; FH7; FQ6; FS4). In fact, conventional roles exhibit a pattern of family arrangements and sharing of tasks that determines this position of “provider” to men and women as home “caretakers” (HL9; HK12; HO13; HW8; FC6; FL8): *Her doing things at home and me bringing money to the house (…)* (HK13).

The social role of women as “caregivers” is recognized by men: *She kept insisting: “Go do the treatment, go after the treatment”. Then I did* (HBB7). Even among women, it is recognized that such a role is their domain: *But I thought that the ones who would give me more support would be the sisters, the women, but I didn’t have the support* (FQ1).

The perception about the sharing of domestic tasks was also observed, by the men, when they affirm that there are household activities that must be carried out by them, such as the repair of objects and equipment and, others, by women, reaffirming its naturalization: *(…) I make repairs, repairs are for men, activity for men, let’s say, repairing a wall, plastering a house, mowing the weeds. But domestic (…) washing the dishes, washing clothes, I’m still from that time, but the problem is that not doing it… If I go wash a glass, I break two…* (HL12).

On the other hand, and in general, the speeches by the women interviewed differ from those of the men with regard to the sharing of domestic tasks, since in almost all reports, they stated that such activities were carried out only by them or with the help of other female figures, who were part of the family nucleus: *Woman, there is no sharing here, (…) I do everything, I wash the dishes, tomorrow I sweep the house* (FC6).

It should be noted that some women reinforce the naturalization of task sharing, as it is understandable for them that men do not support/do not perform domestic activities, on the grounds that men’s work takes place strictly outside the home, considering it is also understood that domestic work does not constitute work activity: *He works at night, during the day he sleeps a little* (FH10); (…) *he works, studies at night, then there’s no way he can help* (FM7). And the same was manifested by some men (HL11; HO13; HT29; HW8): *Domestic activities, it’s my mother who always does it. No, I don’t help, I spend the day working* (HW8; HL11).

With regard to the experience of TB, in some statements there seems to be no difference between participants of both sexes (HA4; HD3; HE2; HY2; HAA24; FF2):

*I think the disease is normal, there is no sex, there is no person; even a child gets sick, the disease has no age, it has no limit* (HE2; FP16).

*Just a sick person, there’s no difference* (HD3).

Nonetheless, some women put themselves as “*helpless*” and, in this sense, more prone to illness (FF3; FK9).

From the men’s perspective, their social role and the stigma caused by TB have an effect on the situation experienced:

*(…) it’s complicated, because we have to hide a little… and because of me being a man, it gets worse, I had to stop working and I couldn’t tell anyone (…)* (HT14).

The bond with the health service is evidenced by listening, by carrying out home visits, by directly supervised treatment, by the understanding and flexibility of the health team during examinations (HL17; HP21; HT39; HW12; HY25; HAA20; HEE28; HFF13; FA7; FC9; FE13; FG15; FY12; FAA8). Professionals’ dialogue and guidance were elements highlighted by men and women as facilitators, as they provide information about the clinical case, the disease, and clarification on the possibility of a cure (HD11; HK17; HR17; HU13; HV13; HAA20; FC9; FD8; FG15; FJ8; FO16).

In addition, the geographic location of the health service, highlighted mainly by men, contributes to the reduction of financial expenses. The fact that the treatment is performed free of charge by the health services and, mainly, the provision of medication, were also admitted as facilities (HC6; HCC19; HV13).

With regard to the elements that condition the treatment of TB, it was found that, for the most part, both groups of interviewees indicated the absence of difficulties, mainly due to the support received from the team of qualified health professionals and as a result of affective bonds – from family members and/or friends (HA7; HB5; HF4; HG3; HH10; HY14; FA7; FA16; FD7; FG15 ; FF13):

*(…) I got it with the help of friends* (HB5).

*I liked the team, everything was good* (HA7).

*Everyone supported me* (HI17)

*She was an excellent doctor (…)* (HJ24)

*Treatment went well (…) I liked the team, it was all good* (HA7).

Those who reported difficulties highlighted the effects caused by the drugs (HF8; FD9; FP16; FQ20), the morning time of taking the medication (HFF14, HW13,15), and the feeling of increased side effects as a result of fasting prior to taking the medication (HFF14; HDD4).

For women, the duration of treatment was identified as a difficulty, but it did not constitute an impediment to therapeutic adherence:

*(…) malaise, I spent six months with a headache, but I couldn’t stop taking the medication* (FC7).

Some men admitted that breaking some habits, such as smoking and drinking (HC1; HJ1; HT5; HQ10), delay in starting treatment due to delayed diagnosis (*I went to the unit several times and they didn’t say what I had* [HT5]), the lack of medicines at the health unit, the bureaucracy to take the drug at the health unit, the wait for Directly Observed Treatment, which became unbearable due to the feeling of hunger, given the need for fasting (HH4; HBB11; HH12; HQ13), constituted challenges for treatment follow-up. The wear and tear suffered by the withdrawal of men from the labor market, with repercussions on the satisfaction of needs for life maintenance, was pointed out as the cause of treatment abandonment (HT2,3,5).

Still on the elements that condition the treatment of TB, they mentioned the difficulty in obtaining some form of governmental social protection. Most of the interviewees from both groups did not get any incentive, which made it difficult to attend consultations and take medication, in a supervised way (FG3; FI24), which can constitute a barrier to treatment adherence:

*I got here, the social worker asked for the benefit, but when I got there, it went wrong, so I didn’t want to go after it anymore (…)* (HDD4).

*“If I had help from the government, it would be better, because I had to go back to work and I couldn’t continue the treatment” (….)* (HDD7)

## DISCUSSION

The study allowed identifying the participants’ sociodemographic characteristics in the study and some aspects related to gender, concretized by social roles, in the treatment of TB.

In relation to the characteristics of people undergoing treatment, level of education, work situation, number of hours spent at home and in extra-domestic activities were significant, expressing, objectively, peculiarities between men and women. Men had lower level of education, were working, worked fewer hours at home and worked in activities demanding more time away from home than women. Such inequality has historically been justified by the naturalized perspective of behavioral differences between the sexes. The organization required for the perpetuation of the species has been generically explained by biological determinism, which imposes the representation of the fragility of women, who would be responsible for lighter tasks, and of masculine robustness, with the responsibility for heavier tasks. In this perspective, the male figure must protect the woman who, in her turn, depends on protection, is responsible for the care of the home and for zeal and procreation activities^(15,16)^. This world view makes up one of the findings of the present study, in which man places himself as the one who is responsible for supporting the family and the woman is responsible for domestic activities.

Women had higher level of education than men, although still low, and they said they spent more time on activities at home. In contemporary times, in many developing countries, and Brazil is an objective example, women have a double or triple workload, taking care of the family and home, besides performing extra-domestic work activities with wages, as a rule, lower than those of men^(17)^, resulting in gender inequality at work. In 2019, for instance, women obtained 77.7% of the amount received by men^(18)^. The impact of TB on these women can therefore be much more severe, not only in the individual dimension, but also affecting their families and society itself^(17)^.

On the other hand, gender issues are directly related to the economic, cultural and social sphere, reiterating that the greater involvement of the disease by men may result from the fact that they are more exposed to the bacillus causing the disease. In fact, the region where the study was carried out still has historical characteristics related to work and the territory that give greater possibility of exposure to interaction, on the part of men, outside the home. It is true that, in many societies, men are financially responsible for guaranteeing the family’s survival conditions, which can lead to contexts in which greater vulnerability takes place^(19)^.

TB is determined by living and working conditions, which contributes to increased social vulnerability. Some studies have reported that inadequate living conditions are particularly precarious among women, who also have less access to economic resources. When they are able to integrate the formal sector, they usually perform subordinate functions that require less qualification than for men, pointing out that such elements also contribute to hindering women’s access to diagnosis and adherence to treatment^(20)^.

The participants’ statements in the present study evidence the reinforcement of the relationships socially imposed on men and women and that, many times, end up being naturalized. To understand the roles played by both groups, it is important to understand that, over time, the spaces occupied were different, with the private one reserved for women and the public one for men. The insertion of women in the private space (at home), sometimes seen as mandatory, being recognized as “housewives”, ends up canceling their desires, making the occupation/presence in public spaces, in politics, in the labor market impossible, and not rarely impairing their autonomy, which reflects on their self-care^(21)^.

Another line of findings refers to the sharing of tasks, which is closely associated with the subjects’ perception of their role in the family. For women, this sharing does not exist, as they seem to understand domestic care as mandatory, restricting it to men only in cases in which the latter can only “help”. In the case of those who work out, they also believe that domestic activities are their own responsibility, which once again naturalizes this representation. The roles of priority dedication to domestic life and family members, reported by the women in the present study, are based on a historically and socially constructed conception^(11)^. This female gender stereotype ends up being naturalized and distinctive; behaviors contrary to this standard would be deviations and, therefore, devalued. Thus, many women sacralize the family to maintain the private sphere, which can compromise their individual identity^(22)^.

This reality, also expressed in the speeches of the women in this study, is reiterated in the limited literature that deals with the relationship between gender and TB. It was observed that the conception of responsibility for the custody of the family still predominates, especially among those who perform the functions of “housewife” and “provider”. These functions made them postpone the search for a TB diagnosis, for fear of having to move away from the family nucleus and that the family members would be left without the one who “watches over everyone”, with repercussions on the household budget, and consequences for adherence to the treatment^(23)^.

On the other hand, in some situations, they showed concern regarding the maintenance of activities at home and family finances, which determined that they sought health care more quickly and aimed the cure to take care of family members again^(24)^.

Women in Brazil, despite having many achievements in the public sphere, still suffer from the prevalence of gender inequality that keeps them in conditions of subordination. This difference is fueled by the sexual division of labor, as can be seen in the National Household Sample Survey (PNAD), which shows that, in the last quarter of 2020, 52.9% of women were unemployed, compared to 47.1% of men^(24,25)^. This can characterize the predominance of women in domestic activities and their undervaluation, since this work is not quantified in the calculation of the social wealth produced, despite being an indispensable task in the construction of life^(26)^.

Such a scenario can be further worsened by the impact of the new coronavirus (SARS-CoV-2) pandemic, with consequences for the distribution of work by gender. Women tend to be predominantly in service sectors that have been subject to lockdowns or social distancing measures. On the other hand, they are also more present in sectors that have been defined as critical to the COVID-19 response. Furthermore, the need to carry out lockdown, in many cities around the world, together with school closures, can further exacerbate gender disparities in childcare and household chores^(27)^. It is important to highlight that the pandemic also had consequences in terms of access to services that provide assistance in tuberculosis, which had to be reallocated to the care of patients with COVID-19, with repercussions on early diagnosis and timely assistance for patients with TB.

As for the perception of TB in everyday life, men seem to seek to reinforce their masculinity, by publicly displaying stoicism, not showing suffering, weakness, or fragility outside the home; however, paradoxically, in the private environment they resorted to women’s care. Men, by admitting that they are stronger to face the disease than women, bet on the conception based on their natural physical structure. Due to this interpretation, many men delay seeking health care, with consequences for treatment adherence^(28)^.

Gender inequality is noted in the TB epidemic worldwide and, among the various processes that evidence this reality, there are socio-cultural-economic differences. The role played by women, in some cultures, hinders their autonomy, reduces their accessibility to medical services, with repercussions on adherence to TB treatment. On the other hand, the roles socially required of men can increase social contact, leaving them more exposed to the bacillus, as mentioned above, and when diagnosed with the disease, they can be fragile. The change in routine, as a result of the disease, can lead to financial damage to them and the whole family, with repercussions on treatment adherence. In fact, some studies report that female and male gender roles can influence patients in treatment adherence, corroborating the findings of the present study^(7,29)^.

Studies related to gender differences and TB show the difficulties that women and men have faced in obtaining the diagnosis and starting the treatment of TB. They point out that women face more barriers than men to start treatment and continue until the cure^(30)^. However, men seem to have greater difficulty in adapting to the therapeutic plan during the recommended time and in changing their habits than women^(28)^. The explanation for this finding lies in the belief in his physical strength, which reinforces his masculinity and resilience. As they occupy the role of family providers, the concern about not being able to respond to this need, as a result of the disease, was also understood, in the present study, as a barrier to adherence to TB treatment.

Differences between men and women confirm distinct social roles, configuring elements of vulnerability that can be aggravated by TB. The study contributes to Nursing care, highlighting the importance that the nursing team, especially the Nurse, understand the patients’ conditions based on the comprehension of their social place, including aspects related to their insertion in social production and reproduction, as they reflect different opportunities for access to health, maintenance of life, and coping with the disease, which can influence the process of adherence to treatment.

As limitations of the study, it was found that, quite possibly as a result of its design, it was not possible for aspects to emerge that could deepen the discussion of the gender theme, from the perspective of its participants, reducing the possibility that they could reflect on their social role. Another limitation is the scarcity of studies discussing the health-disease process from a gender perspective and not just in relation to differences in the experience of tuberculosis in males and females.

## CONCLUSION

The observation of the elements related to some individual characteristics and the health-disease process of each group of study participants was made possible.

Men’s social role as financial providers, and women’s as caregivers, are significant elements in the influence of gender on adherence to TB treatment. It is believed that the present study fills a gap in this area of knowledge, by providing health workers with tools related to gender differences in TB control, more specifically, improving adherence to treatment based on these particularities. Although common aspects between men and women in coping with the disease have been found, it is understood that adherence is linked to social reproduction, which, in the same way, can be influenced by gender issues.

However, the interpretation of the findings cannot be generalized, and the importance of carrying out further studies whose object is the analysis from a gender perspective shall be emphasized. On the other hand, it is believed that the findings may also contribute to raising, in health professionals, reflections on gender differences in the health-disease process, with an impact on improved adherence to treatment.
